# Passive versus active educational interventions for nevus and melanoma classification: A randomized controlled study

**DOI:** 10.1111/jdv.20649

**Published:** 2025-03-21

**Authors:** M. Spadafora, R. Pampena, K. Peris, L. Del Regno, L. Cornacchia, M. C. Fargnoli, C. Pellegrini, P. Quaglino, S. Ribero, P. G. Calzavara‐Pinton, M. C. Arisi, M. Mirra, M. Raucci, A. Fusco, S. Kaleci, J. Chester, G. Pellacani, C. Longo

**Affiliations:** ^1^ Azienda Unità Sanitaria Locale – IRCCS di Reggio Emilia, Skin Cancer Center Reggio Emilia Italy; ^2^ Clinical and Experimental Medicine PhD Program University of Modena and Reggio Emilia Modena Italy; ^3^ Private Practice Formia Italy; ^4^ Department of Translational Medicine and Surgery, IRCCS A. Gemelli Sacred Heart Catholic University Rome Italy; ^5^ Dermatology, Dipartimento di Medicina e Chirurgia Traslazionale Sacred Heart Catholic University Rome Italy; ^6^ Department of Biotechnological and Applied Clinical Sciences University of L'Aquila L'Aquila Italy; ^7^ Dermatologic Clinic, Department of Medical Sciences University of Turin Turin Italy; ^8^ Department of Dermatology University of Brescia Brescia Italy; ^9^ Department of Surgery, Medicine, Dental Medicine and Morphological Sciences University of Modena and Reggio Emilia Modena Italy; ^10^ Dermatology Clinic, Department of Clinical Internal, Anesthesiological and Cardiovascular Sciences Sapienza University of Rome Rome Italy; ^11^ Department of Dermatology University of Modena and Reggio Emilia Modena Italy

## Abstract

**Backgrounds:**

Patients or family members first notice around 50% of primary melanoma lesions. Targeted educational campaigns for non‐medical individuals improve melanoma detection rates, but the most effective initiatives are unclear.

**Objectives:**

To compare the efficacy of passive versus active intervention for non‐medical individuals in classifying nevi and melanomas.

**Methods:**

A multicentric randomized controlled study randomly assigned subjects to receive active intervention (a dermatologist explaining basic rules for melanoma detection) or passive intervention (subject independently reading the basic rules). Subjects were asked to classify 60 clinical photos of nevi and melanomas as ‘at risk’ of malignancy and nominated the rule(s) they applied at 3 time points—before (T0), immediately following (T1), and 30 ± 2 days after (T2) the educational intervention.

**Results:**

We randomized 364 patients. We included in the analysis 336 subjects (female 61.3%, 156 in the passive and 180 in the active intervention group) with a mean age of 44.5 years. Overall, detection rates of lesions ‘at risk’ improved from 71.2% (T0) to 86.4% (T1). At T2, detection rates were significantly higher after active intervention (83.7% vs. 86.8%, *p* = 0.017). Although an overall improvement was described after both interventions, rates of correct responses according to lesion‐specific features were significantly higher in the active intervention group for lesions that met ugly duckling (UD) rule criteria at T1 and both rules (ABCDE and UD rules) criteria at T2. Correct (full or partial) rule applications were observed in 80% at T1 (40.2% and 38.4%, respectively) and at T2 (40.4% and 37.8%, respectively), with significantly higher correct rule application in the active group at T1 (*p* = 0.001) and T2 (*p* = 0.03).

**Conclusions:**

Active educational intervention is more effective than passive education in improving nevi and melanomas classification and correct rule application in non‐medical individuals, with stable performance observed over time.

## Graphical abstract

This graphical abstract synthesizes the outcomes of the educational intervention aimed at improving the application of the ABCDE and Ugly Duckling (UD) rules in the assessment of melanocytic lesions in non‐medical people.

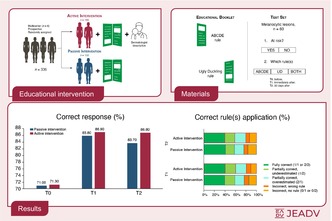

**Why was the study undertaken?**

Educational campaigns targeting non‐medical individuals, usually the first to detect atypical melanocytic lesions, could improve the identification of suspicious lesions, although the most effective educational strategies remain uncertain. Open access publishing facilitated by Universita degli Studi di Modena e Reggio Emilia, as part of the Wiley ‐ CRUI‐CARE agreement.

**What does this study add?**

Active educational interventions, which consist of a standardized explanation by a dermatologist of melanoma lesion detection, increase the ability of non‐medical individuals to assess lesions at risk.

**What are the implications of this study for disease understanding and/or clinical care?**

From our study, subjects who received an active educational intervention were more likely to correctly identify lesions at risk of malignancy, apply the correct rule for lesion identification, and retain useful information through time. The most effective lesion assessment by non‐medical individuals is based on lesional‐specific features (ABCDE rule) and can be enhanced with a comparative approach (ugly duckling rule).

## INTRODUCTION

Early diagnosis followed by surgical excision represents the most effective treatment for melanoma.^1^

It has been found that from 41% to 57% of primary melanoma lesions are initially suspected by patients or family members following visual inspection.^2^, ^3^ It is hypothesized that campaigns among non‐medical individuals about the characteristics of atypical melanocytic lesions may increase early melanoma detection rates. The ABCD rule, encompassing Asymmetry, irregular Borders, Colour variegation, and Diameter >6 mm, was established in 1985 to identify independent features suggesting the risk of malignancy. In 2004, the rule was updated to include the letter E for Evolution,^4^, ^5^ where features of the ABCD rule are modified over a short time period.

In patients with many atypical lesions, the application of the ABCDE rule is considered to overestimate the risk of malignancy.^6^ Hence, the use of the ‘ugly duckling’ (UD) sign, an additional clinical rule to determine the most melanocytic atypical lesions from common nevi.^7^ A recent study showed how the UD sign can enhance the effectiveness of the ABCDE rule in helping non‐medical individuals recognize melanoma.^8^ However, the most effective educational initiative for non‐medical individuals to recognize suspicious lesions is unclear.

This randomized controlled study aimed to compare the efficacy of passive (a booklet explaining ABCDE and UD rules) versus active (the same booklet explained by a dermatologist) intervention for non‐medical individuals in correctly identifying atypical skin melanocytic lesions (melanoma and nevus). The primary outcome was to analyse the proportion of correct responses between groups before and after the test administration, and whether knowledge was retained. The secondary outcome was to evaluate the application rates of the correct clinical rule to classify a lesion.

## MATERIALS AND METHODS

### Study design and patient enrollment

A randomized multicentric controlled study of active versus passive educational intervention was developed at the Skin Cancer Center of ASL/IRCCS Arcispedale Santa Maria Nuova in Reggio Emilia, Italy. Active interventions comprised the delivery of educational material accompanied by a standardized explanation of the booklet information from a dedicated dermatologist. Passive interventions specified the delivery of the educational material only, without any additional explanation.

Subjects were recruited with a competitive enrollment strategy at 6 dermatology centres in North‐central Italy (Skin Cancer Center of ASL/IRCCS Arcispedale Santa Maria Nuova in Reggio Emilia, Dermatology Unit of Azienda Ospealiero‐Universitaria Policlinico of Modena, Dermatology Unit).

AOU Città della Salute e della Scienza di Torino, Dermatology Unit – University and ASST‐Spedali Civili Hospital of Brescia, Department of Dermatology Sacred Heart Catholic University in Rome, Dermatology Unit – San Salvatore Hospital of L'Aquila. Study inclusion comprised subjects attending an initial dermatological skin examination at any of the 6 participating centres who signed specific informed, written consent to enrollment and completed the baseline test, between January 2019 and December 2020. Criteria specified the exclusion of subjects with personal or familial history of melanoma and/or familiarity with at least one of the tested rules (ABCDE and UD). The study was conducted according to the Principles of Helsinki, General Data Protection Regulation (GDPR) and all patients whose images were included in the test set, gave written informed consent to image publication. The study was approved by the local ethics committee (EC:184/2019/SPER/IRCCSRE) and was registered on ClinicalTrials.gov with the ID NCT04507048.

The educational intervention included the development of a patient brochure outlining two clinical rules of atypical melanocytic lesion identification: the ABCDE and UD rules. Both rules were described with written comments accompanied by clinical images for each letter of the acronym (Figure S1), and in the case of the UD rule, a clinical photo of a patient's back with suspicious lesions indicated (arrows) was accompanied by close‐up clinical images of the suspicious nevi (Figure S2).

### Testing

Educational interventions were performed by testing the non‐medical individuals' ability to correctly identify lesions' risk of malignancy from a test set. A test set was created by 2 dermatologists experienced in dermato‐oncology (RP and CL) (Figure S3). A total of 60 clinical photos of melanocytic lesions (with limited demographic, lesion and evolution information) were selected to facilitate the straightforward identification of features from the ABCDE and UD rules. The test set included nevi e melanomas only, as the ABCDE and UD rules are usually applied to differentiate these lesions in the clinical setting. Other lesion types were, therefore, not represented in the test‐set. Images were collected from the Skin Cancer Center database at Arcispedale Santa Maria Nuovo in Reggio Emilia. The test was made up of two image subsets; stratified according to lesion risk—‘at risk’ or ‘not at risk’ of malignancy. Images were randomly presented. The test set comprised 30 lesions ‘at risk’ (9 melanomas and 21 nevi) and 30 ‘not at risk’ of malignancy. For each lesion classified ‘at risk’, the expert dermatologist nominated the appropriate rule according to lesion presentation; ABCDE (*n* = 10), UD (*n* = 10) or both (*n* = 10).

### Educational intervention

Subject demographic data were collected at T0 by the investigator before the administration of the educational intervention, using a case report form (CRF). Subjects were randomly assigned (~1:1 at each center) to receive either intervention.

To minimize bias, a nurse provided the booklet to enrolled subjects in both groups. Active intervention was when an additional, individual patient education session was administered by a dedicated dermatologist to each patient. The dermatologist followed a pre‐designed and harmonized lesson to explain the content of the booklet only. In both the intervention groups (immediately following the dermatological consultation), subjects were invited to read the booklet (15 min unsupervised) before completing the test for a second time (T1).

### Assessment

Subjects were instructed to (1) classify each lesion as ‘at risk’ when suspicious of melanoma or ‘not at risk’ when not suspicious of melanoma and (2) to nominate the rule(s) applied when the lesion was classified ‘at risk’. Patients were tested at 3 time points: before (T0), immediately following (T1), and 30 ± 2 days after (T2) the educational intervention. Outcomes were blindly assessed by a dermatologist (R.P.).

Subject responses were interpreted according to (i) lesion risk assessment (all lesions and lesions ‘at risk of melanoma’), (ii) lesion risk assessment stratified by overall lesion presentation (rule(s) applied), (iii) correct rule application. A correct response of lesion risk assessment was considered when the subject classified a lesion ‘at risk of melanoma’ in accordance with the pre‐testing classification attributed by the expert dermatologist. The application of a rule was considered correct when the subject selected the same clinical rule for lesion identification as indicated at pre‐testing by the expert dermatologist.

### Statistical analysis

Statistical analysis was performed using STATA® software version 17 (StataCorp^9^). Descriptive statistics summarized baseline demographic and clinical characteristics (entire cohort and intervention groups).

For binary outcomes (e.g. correct vs. incorrect lesion classification), data were summarized as frequencies (*n*, percentage [%]) and comparisons between groups were performed using the Pearson's chi‐square test. Continuous variables (e.g. age) were presented as mean and standard deviation (SD). Group comparisons were analysed with the unpaired Student's *t* test or the Mann–Whitney *U* test.

To assess changes over time within groups (e.g. between T0, T1, and T2), McNemar's test (for paired binary outcomes) and paired *t*‐tests (for continuous outcomes) were used. A *p* value <0.05 was considered statistically significant.

## RESULTS

We randomized 364 patients. We included in the analysis 336 subjects (female 61.3%), who completed the baseline test after enrolment, with a mean age of 44.5 ± 14.9 years (range 17–80 years). The passive intervention group consisted of 156 subjects (mean age 44.7 ± 14.9, female 62.2%), and the active intervention group consisted of 180 subjects (mean age 44.5 ± 15.1, female 69.9%) (Table 1 and Figure S4) Question response rates were complete (100.0%) at T0, 90.7% at T1, and 30.1% at T2.

**TABLE 1:** Demographic subject data.

	Total	Intervention type		*p* value
		Passive	Active	
	*n* = 336	*n* = 156	*n* = 180	
	(100.0%)	(46.4%)	(53.6%)	
Age, mean ± SD	44.5 ± 14.9	44.7 ± 14.9	44.4 ± 15.1	0.845
(range)	(17–80)	(18–75)	(17.80)	
Gender, *n* (%)				
Male	127 (37.8)	58 (37.2)	69 (38.3)	0.752
Female	206 (61.3)	97 (62.2)	109 (69.6)	
Missing	3 (0.9)	1 (0.6)	2 (1.1)	

Abbreviation: SD, standard deviation.

### Lesion risk assessment

**FIGURE 1:**
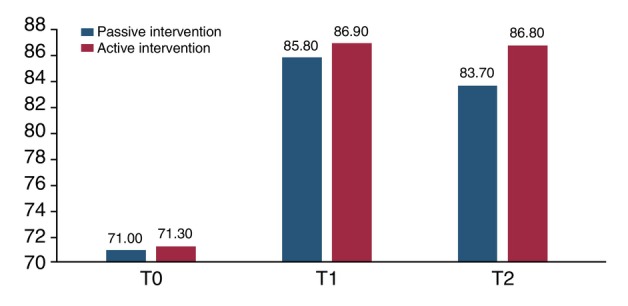
Diagram representation of correct responses in the two intervention groups (passive vs. active) divided by the times of test collection (T0–T2).

In Table 2 and Figure 1, we report the rates of correct responses at each time point, stratified according to intervention group. A sub‐analysis was performed considering responses on cases at risk for melanoma according to the ABCDE and/or UD rules, as assessed by the dermatologists during the development of the image set. At T0, correct overall answer rates were 71.2%, with an insignificant difference according to passive or active interventions (71.0% vs. 71.3%). At T1, the correct overall answer rates improved to 86.4%. A one‐point comparative improvement was noted within the active intervention group (85.8% vs. 86.9%). At T2, the correct answer rates remained stable with a significant difference between the intervention groups, in favour of the active intervention group (83.7% vs. 86.8%; *p* = 0.017).

**TABLE 2:** Rates of correct responses at each time point, stratified according to the intervention group.

Time	Responses			
	Received	Correct^a^		
	Total	Total	Passive	Active
	*n* (%)	*n* (%)	*n* (%)	*n* (%)
T0	20,160 (100.0)	6936/9744 (71.2)	3214/4525 (71.0)	3722/5220 (71.3)
T1	18,299 (90.7)	7649/8844 (86.4)	3436/4002 (85.8)	4213/4842 (86.9)
T2*	6060 (30.1)	2502/2929 (85.2)	1092/1305 (83.7)	1410/1624 (86.8)

^a^ Correct responses classified was ‘at risk for melanoma’ according to the ABCDE and/or UD rules as assessed by the dermatologists.

* *p* value = 0.017.

### Lesion risk assessment stratified by overall lesion presentations

**FIGURE 2:**
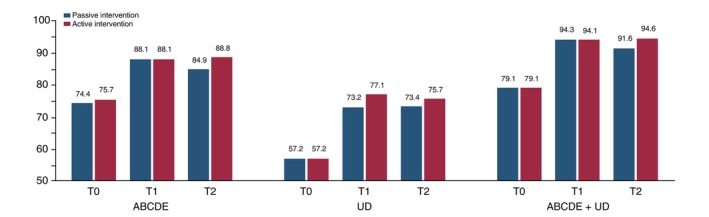
Diagram representation of correct identification of lesion ‘at risk’ stratified for the rule (ABCDE rule, ugly duckling rule, or both, as indicated by dermatologists) and subdivided by intervention groups (passive vs. active) and by time of test collection (T0–T2).

In Table 3 and Figure 2, we reported the rates of correct identification of lesions ‘at risk’ according to lesion presentation (as indicated by the correct rule of ABCD rule, UD rule, or both). Throughout the study period, patients' performance improved. Specifically, the identification of atypical features among presented lesions (‘ABCDE’ rule) improved from 75.1% at T0 to 88.1% at T1 and 87.0% at T2. The frequency of correct responses was higher in the active intervention group. The identification of the worst lesion (‘UD’ rule) improved from 55.0% at T0 to 75.1% at T1 and 73.1% at T2. Again, the frequency of correct responses was higher in the active intervention group and statistically higher at T1 (*p* = 0.012). The identification of atypical features in the worst lesion (the lesion met criteria for both ‘ABCDE’ and ‘UD’ rules) improved from 79.4% at T0 to 94.2% at T1 and 92.9% at T2. The frequency of correct responses was similar in the intervention groups at T0 and T1 but was significantly higher in the active intervention group at T2 (*p* = 0.039).

**TABLE 3:** Rates of correct identification of lesions ‘at risk’ according to lesion presentation (as indicated by ABCD rule, UD rule, or both) subdivided into different intervention groups at each time point.

Rule, time point	Total	Intervention type	
		Passive	Active
	*n (%)*	*n* (%)	*n* (%)
ABCDE			
T0	2522 (75.1)	1160 (74.4)	1362 (75.7)
T1	2688 (88.1)	1216 (88.1)	1472 (88.1)
T2	879 (87.0)	382 (84.9)	497 (88.8)
UD			
T0	1478 (55.0)	687 (57.2)	791 (54.9)
T1*	1799 (75.1)	787 (71.3)	1012 (75.7)
T2	591 (73.1)	259 (71.9)	332 (74.1)
ABCDE + UD			
T0	2936 (79.4)	1367 (79.7)	1569 (79.2)
T1	3162 (94.2)	1433 (94.4)	1729 (94.1)
T2**	1032 (92.9)	451 (91.1)	581 (94.3)

Abbreviation: UD, ugly duckling.

*p value = 0.012, **p value = 0.039.

### Correct rule application

**FIGURE 3:**
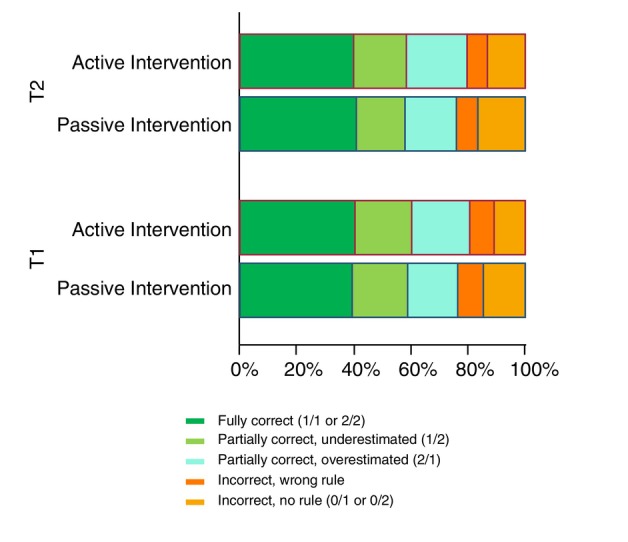
Diagram representation of correct rule(s) application in the intervention groups (passive vs. active) subdivided by the time of test collections (T1, T2).

As shown in Table 4 and Figure 3, around 80% of the overall rule applications were fully or partially correct at T1 (fully correct 40.2% and 38.4%, respectively) at T2 (fully correct 40.4% and 37.8%, respectively). Between T1 and T2, the frequency of the incorrect rule decreased (from 8.7% to 7.2%) but the absence of a rule when it should have been applied worsened (from 12.5% to 14.6%). The active group reported significantly better correct rule application (fully or partially) at both T1 (*p* = 0.001) and T2 (*p* = 0.03).

**TABLE 4:** Rates of correct rule(s) application, stratified for the time (T1 and T2) in different intervention groups.

Rule application	T1				T2			
	Total	Passive, *n* (%)	Active, *n* (%)	*p* value	Total	Passive, *n* (%)	Active, *n* (%)	*p* value
Fully correct (1/1 or 2/2)	3553 (40.2)	1588 (39.7)	1965 (40.6)	<0.001	1182 (40.4)	534 (40.9)	648 (39.9)	0.03
Partially correct								
Underestimated (1 of 2)	1711 (19.3)	765 (19.1)	946 (19.5)		520 (17.7)	222 (17.0)	298 (18.3)	
Overestimated (2 instead of 1)	1697 (19.1)	706 (17.6)	991 (20.4)		589 (20.1)	237 (18.2)	353 (21.6)	
Incorrect								
Wrong rule	774 (8.7)	350 (8.7)	424 (8.7)		211 (7.2)	99 (7.6)	112 (6.9)	
No rule (0/1 or 0/2)	1110 (12.5)	593 (14.8)	517 (10.7)		427 (14.6)	213 (16.3)	214 (13.2)	

## DISCUSSION

Our study proves that active educational interventions, compared to passive educational interventions, increase the likelihood of non‐medical individuals correctly classifying melanocytic lesions through the application of the most appropriate ABCDE and/or UD rules. Non‐medical individuals are also more likely to correctly identify lesions at risk of malignancy when they have more atypical features, requiring the ABCDE rule or the ABCDE and UD rules together.

Early diagnosis is crucial in reducing melanoma‐associated mortality and morbidity,^1^, ^10^ and the detection of lesions at risk by patients and their family members has proven to positively influence early consultations.^4^, ^11^ Conversely, patient delay in seeking medical care has been claimed to be the main factor leading to melanoma‐related deaths.^12^ Delay has been linked to factors such as low patient educational levels, living alone, and poor melanoma awareness.^12^, ^13^ Many authors have called for more efforts in the promotion of public‐oriented educational interventions to shorten the delay between melanoma appearance and patient presentation.^2^, ^14^, ^15^, ^16^

There is an ongoing debate about the best way to offer educational interventions. To date, only a few studies have compared various strategies to enhance the ability of non‐medical individuals to detect lesions at risk. In 2004, Oliveira et al.^17^ conducted a randomized trial on patients with multiple atypical nevi. The study found that people who received total body photographs were more likely to regularly perform self‐skin checks. In 2014, Jauda et al.^18^ demonstrated that patients who received both video and written information were more likely to perform self‐skin examinations compared to those who only received a written booklet. However, Maganty et al.^19^ showed that using a game‐based educational approach was just as effective as written information in helping non‐medical individuals recognize melanoma features.

Our study proves that lesion risk assessment, either associated with a passive or active intervention style, is improved among non‐medical individuals. Overall, the rate of accurate answers to the question ‘Is this lesion at risk for melanoma?’ was approximately 15% higher at T1 compared to T0. In the setting of skin cancer early detection, where prompt identification is crucial, even a small percentage improvement might lead to substantial benefits at a population level with a potential high clinical relevance.

This study also reports an improvement in patients' performance following the active intervention by a health professional in explaining the educational material. Similar outcomes have previously been described in a setting where the medical professional was a dedicated nurse.^17^ As this study was conducted over the COVID‐19 pandemic, the number of participants at T2 was greatly reduced, introducing a potential attrition bias. However, the overall rate of correct answers at T2 improved greatly compared to T0, thanks to the educational interventions. Active intervention was associated with higher correct identification of lesions at risk, with a stronger retention of information compared to subjects who received a passive intervention approach.

Lesion risk assessment stratified by overall lesion presentation was assessed through the application of the correct rule (ABCDE, UD, or both) in identifying lesions at risk. The ABCDE rule assists in identifying atypical melanocytic lesions, assessing the individual lesion for various features, compared to the UD rule, which relies upon a comparative approach with other lesions on the same body area. Prior to and following educational intervention, the lowest performance in lesion detection was observed among lesions that should have been identified with the UD rule. Comparatively, the highest detection performance was noted among lesions that met both ABCDE and UD rules, that is, lesions with many and/or highly accentuated atypical features compared to close‐by nevi. These results support an assumption that patients are less able to detect lesions at risk with a comparative approach only, whilst the most effective lesion assessment relies on specific features (ABCDE rue) and can be enhanced with a comparative approach (UD rule). Similarly, Ilyas et al.^8^ described the additional role of UD in enhancing ABCDE rule effectiveness.

Finally, correct rule application was found in our study to be significantly improved among subjects assigned to the active educational intervention arm at T1 and T2. Non‐medical individuals were more likely to fully or partially apply the appropriate rule correctly to lesion assessments when an active intervention was applied.

### Limitations

Our study included patients seeking consultations in dermatological centres for skin assessment and therefore has an inherent patient selection bias and does not represent the general population. Although preserving the unpredictability of allocation, the competitive strategy of enrollment generated a discrepancy in sample size for the intervention groups. Our study outcomes contribute to the general knowledge of the most appropriate educational approaches, but their applicability in busy dermatological centres is limited by obvious time constraints in real‐world scenarios. Moreover, the potential attrition bias caused by the reduced number of participants at T2 should be taken into account. Future research should focus on identifying categories of subjects who may benefit most from active educational interventions.

## CONCLUSIONS

Our study proves that active educational interventions for non‐medical individuals improve nevi and melanoma classification compared to passive educational interventions. Subjects who received an active educational intervention were more likely to correctly identify lesions at risk of malignancy, apply the correct rule for lesion identification, and retain useful information through time. The most effective lesion assessment by non‐medical individuals seems to be based on specific features (ABCDE rule) and can be enhanced with a comparative approach (UD rule).

## AUTHOR CONTRIBUTIONS

Conceptualization: C Longo; R Pampena; data curation: M Spadafora, R Pampena, M Mirra, M Raucci, C Pellegrini, L Cornacchia, S Ribero, MC Arisi, L Del Regno, A Fusco; formal analysis: S Kaleci; supervision: C Longo; investigation: M Spadafora, R Pampena, M Mirra, M Raucci, C Pellegrini, L Cornacchia, S Ribero, MC Arisi, L Del Regno; writing—original draft: M Spadafora, J Chester; writing—review and editing: J Chester, Caterina Longo, G Pellacani, K Peris, P Quaglino, P G Calzavar‐Pinton, MC Fargnoli.

## FUNDING INFORMATION

This study was supported by a grant from SiDeMaST.

## CONFLICT OF INTEREST STATEMENT

None declared.

## ETHICAL APPROVAL

This study was approved by the Institutional Review Board of Azienda Unità Sanitaria Locale—IRCCS di Reggio Emilia, Italy (CE: 184/2019/SPER/IRCCSRE).

## ETHICS STATEMENT

All procedures performed in this study were in accordance with the ethical standards of the institutional and national research committee and with the 1964 Helsinki Declaration and its later amendments or comparable ethical standards. The patients in this manuscript have given written informed consent to the publication of their case details.

## Supporting information


Data S1

## Data Availability

The data that support the findings of this study are available from the corresponding author upon reasonable request.
