# Supplementary material for: Passive versus active educational interventions for nevus and melanoma classification: A randomized controlled study
Source: J Eur Acad Dermatol Venereol. 2025 Mar 21;40(1):109–15. doi: 10.1111/jdv.20649 (PMC12723570; doi:10.1111/jdv.20649)
Supplement: Supplementary file 1 — Data S1 [file JDV-40-109-s001.docx]

**SUPPLEMENTARY**


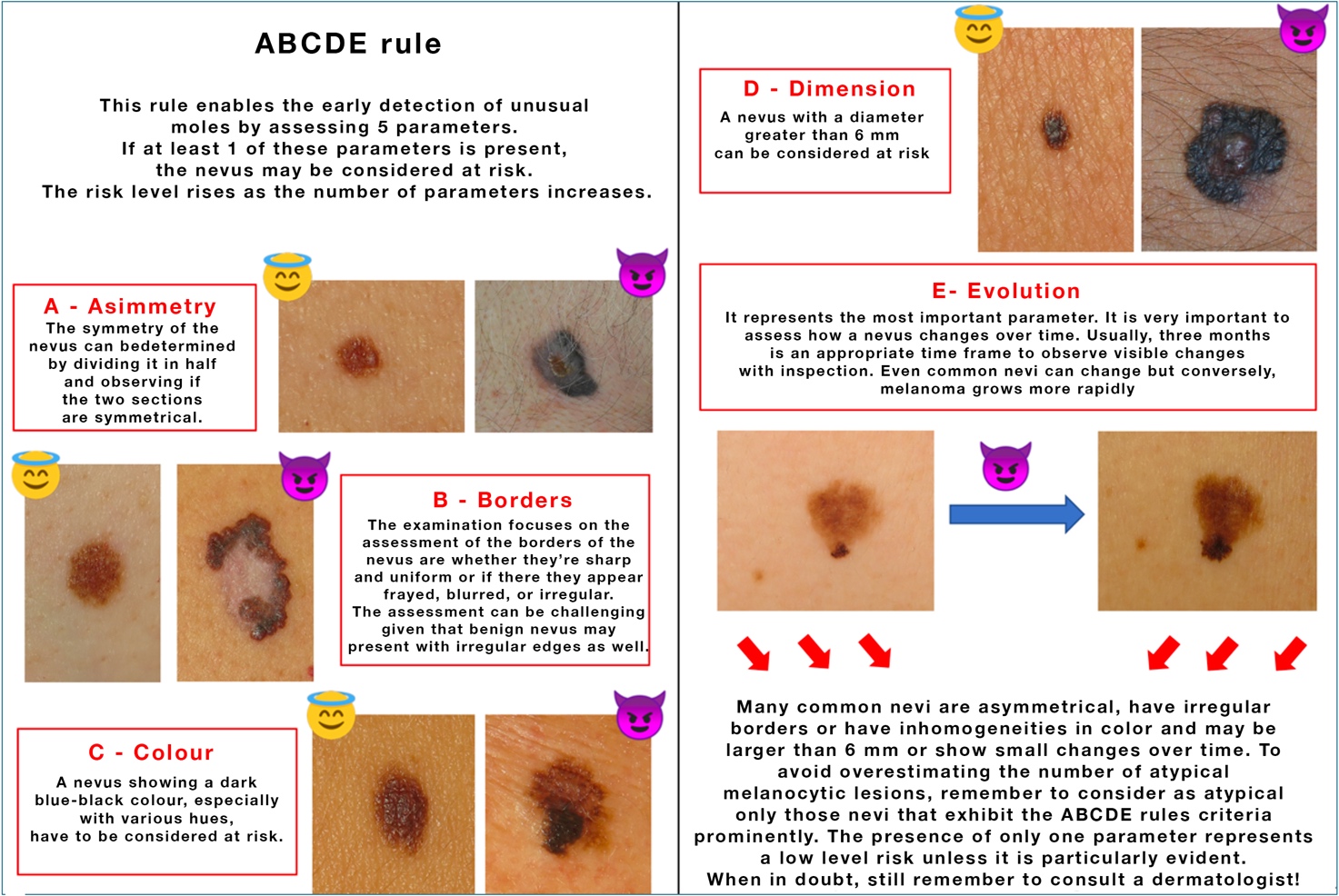


eFigure 1 – ABCDE rule – Insets with written descriptions of acronym letter meanings. Next to each description, we reported images explaining the rule.


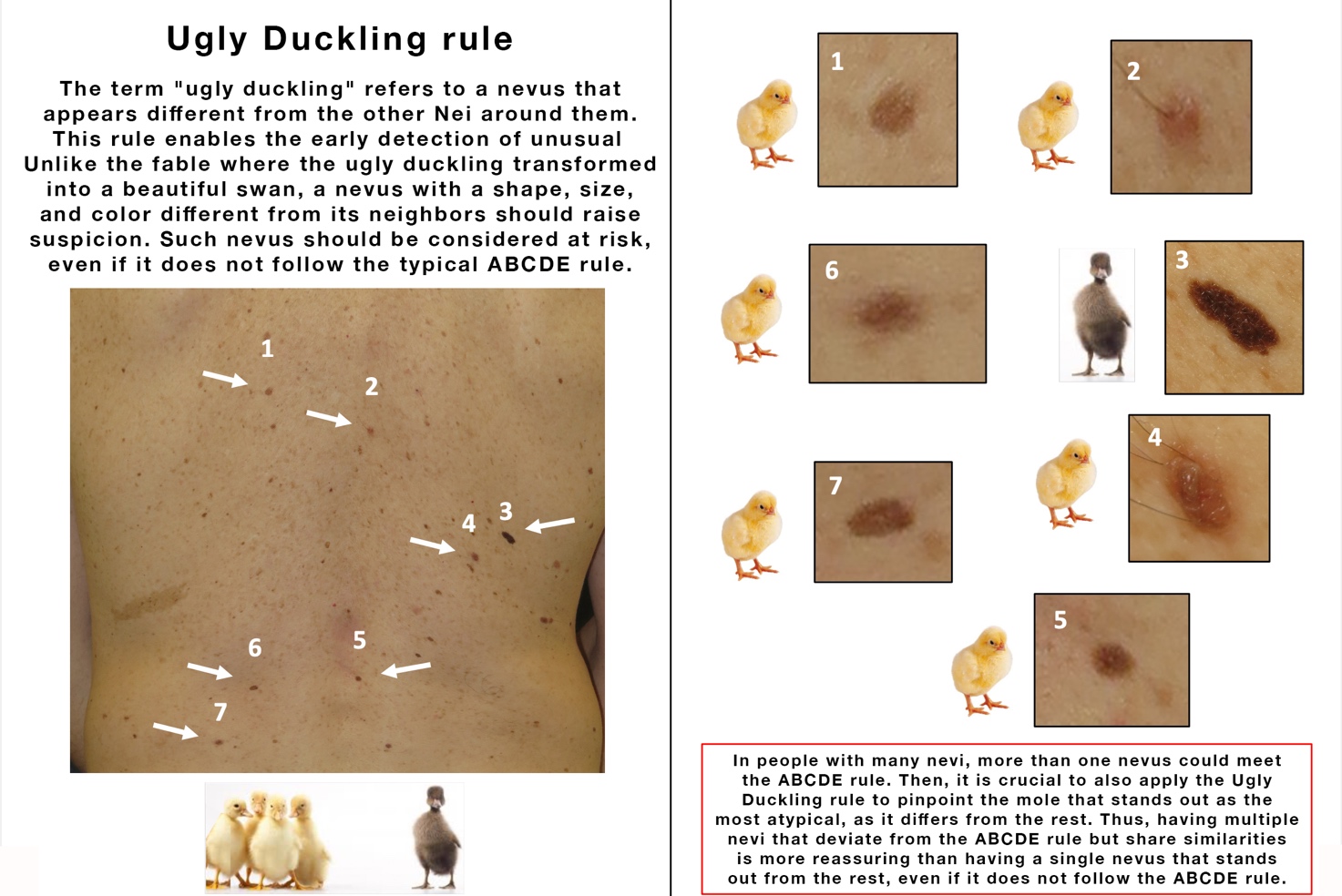


eFigure 2 – Clinical overview of the back of a subject with a detectable melanoma using the "ugly duckling" rule, and a close-up of more relevant nevi, including the atypical one (lesion number 3).


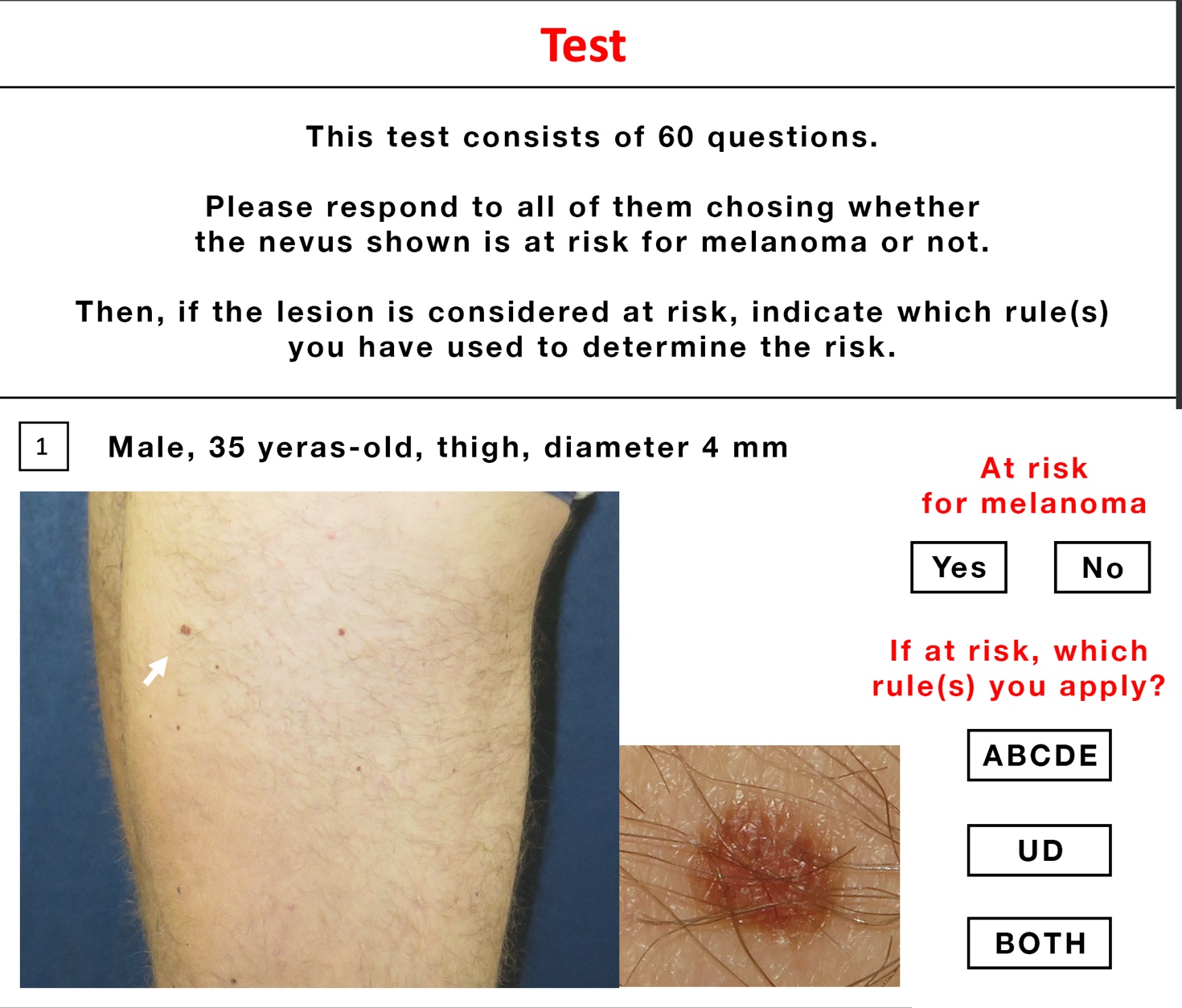


eFigure 3 – Introduction to the test followed by the first case. Each case was presented with relevant features such as gender, age, anatomical site, and size of the lesion. The image on the left shows the anatomical area with an arrow pointing to the tested lesion while the image on the right shows a close-up of the same lesion. On the right side of the page, questions are displayed.

eFigure 4 – CONSORT Flow Diagram

## Analysis

## Follow-Up

## Allocation

Analysed:

n=180 at T0 (10,800 evaluations)

n=167 at T1 (10,020 evaluations)

n= 56 at T2 (3,360 evaluations)

Active Intervention (n=180) at T0

Lost to follow-up:

T2 [CoVid-19 Pandemic] (n=124)

Passive intervention (n=156) at T0

Lost to follow-up:

T2 [CoVid-19 Pandemic] (n=111)

Analysed:

n=156 at T0 (9,360evaluation)

n=138 at T1 (8,280 evaluation)

n= 45 at T2 (2,700 evaluation)

Allocated to Active intervention (n=182)

- Received Active intervention Patients n=180 at T0 (10800 evaluation)
- Withdrawal (n=2) [not completed

Allocated to Passive intervention (n=182)

- Received Passive intervention Patients n=156 at T0 (9360 evaluation)
- Withdrawal (n=26)

Excluded (n=4)

- Decline to participate

## Enrollment

Assessed for eligibility (n=368)

Randomized (n=364)
